# Management of acute coronary syndrome in emergency departments: a cross sectional multicenter study (Tunisia)

**DOI:** 10.1186/s12873-018-0201-6

**Published:** 2018-12-03

**Authors:** Asma Sriha Belguith, Kaouthar Beltaief, Mohamed Amine Msolli, Wahid Bouida, Hela Abroug, Manel Ben Fredj, Imen Zemni, Mohamed Habib Grissa, Hamdi Boubaker, ESCor Investigators group, Mohamed Hsairi, Samir Nouira, On behalf of the ESCorT Investigators group, Krid Jamel, Chtara Majed, Nouira Semir, Marghelli Soudani, Slim Anis, Gassab Khaled, Ben Amor Mehdi, Boukef Riadh, Methammem Mehdi, Aissa Jalled Ahlem, Bouhamed Chafiaa, Borsali Falfoul, Ben Cheikh Maamoun, Souissi Mohamed Hedi, Souissi Sami

**Affiliations:** 1 Epidemiology and Preventive Medicine Department, University Hospital of Monastir, Monastir, Tunisia; 2 Research Laboratory (LR12SP18), University of Monastir, Monastir, Tunisia; 3 National Institute of Public Health, Tunis, Tunisia

**Keywords:** Acute coronary syndrome, Epidemiology, Cardiovascular risk factors, Management, Tunisia

## Abstract

**Background:**

We aimed to describe diagnosed acute coronary syndrome (ACS) and its care management and outcomes in emergency departments (EDs) and to determine related cardiovascular risk factors (CVRFs).

**Methods:**

We conducted a cross sectional multicenter study that included 1173 adults admitted to EDs for acute chest pain (ACP) in 2015 at 14 sites in Tunisia. Data included patients’ baseline characteristics, diagnosis, treatment and output.

**Results:**

ACS represented 49.7% of non-traumatic chest pain [95% CI: 46.7–52.6]; 74.2% of ACS cases were unstable angina/non-ST-segment-elevation myocardial infarction (UA/NSTEMI). Males represented 67.4% of patients with ACS (*p* < 0.001). The median age was 60 years (IQR 52–70). Emergency medical service transportation was used in 11.9% of cases. The median duration between chest pain onset and ED arrival was two hours (Inter quartile ranges (IQR) 2–4 h). The age-standardized prevalence rate was 69.9/100,000 PY; the rate was 96.24 in men and 43.7 in women. In the multivariable analysis, CVRFs related to ST segment elevation myocardial infarction were age correlated to sex and active smoking. CVRFs related to UA/NSTEMI were age correlated to sex, familial and personal vascular history and type 2 diabetes. We reported 27 cases of major adverse cardiovascular events (20.0%) in patients with STEMI and 36 in patients with UA/NSTEMI (9.1%).

**Conclusion:**

Half of the patients consulting EDs with ACP had ACS. Emergency medical service transportation calls were rare. Management delays were acceptable. The risk of developing an UA/NSTEMI was equal to the number of CVRFs + 1. To improve patient outcomes, it is necessary to increase adherence to international management guidelines.

## Background

### Background/rationale

The majority of cardiovascular disease (CVD) deaths occurred in developing countries in 2015 [1], with increasing trends, despite improvements in preventive actions and management [2, 3]. Therefore, acute chest pain (ACP) is a major emergency.

Physicians must quickly recognize highly suspected acute coronary syndrome (ACS) [4–8]. The prevalence of ACS varies within regions in Tunisia, according to the level of urbanization and lifestyle habits. Data on ACS epidemiology and management are rare; therefore, studying ACS in Tunisian emergency departments is necessary.

### Objectives

This study aimed to describe the ACS prevalence and management in Tunisian emergency departments (EDs), to quantify the relationship between cardiovascular risk factors (CVRFS) and ACS, and to determine Major Adverse Cardiac Events (MACE) related to ACS.

## Methods

### Study Design

We conducted a multicenter cross-sectional study that included fourteen EDs.

Data were prospectively collected from February to September 2015.

### Setting

This study included eight university emergency departments (Monastir, Mahdia, Sahloul, Farhat Hached, Kairouan, Rabta, and Ben Arous) and seven regional emergency departments (Mahres, Djebeniana, Jemmal, Ksar_Hellal, Nefidha, Moknine, and ElKram). Tunisia is a Northwest African state covering 165,000 square kilometers. Tunisia's population was estimated to be more than 11 million in 2014. In 2009, there were 12 physicians and 33 nurses per 10,000 inhabitants (inh).

### Participants

We included patients at least 30 years old admitted to the ED for nontraumatic chest pain and who signed an informed consent form. We did not include patients with obvious pulmonary disease or with traumatic chest pain or patients who were unable to give consent (e.g., cognitive impairment) or who were participating in another trial. Deaths at home following suspected ACS were not included. Patients with nontraumatic chest pain and who had diseases other than ACS were considered as the population control.

### Variables

Data included variables related to socio-demographic population characteristics (age, sex, and origin), timeline (chest pain onset according to patient description, ED arrival, ACS treatment start time and length of stay), history (vascular family history, vascular personal history, coronary personal history, revascularization, peripheral arterial history, and stroke) and conventional CVRFs (treated HTA, treated type 2 diabetes, active smoking, and treated dyslipidemia). We also collected information about current medications (beta-blockers, converting enzyme inhibitors (ACE inhibitors), oral antidiabetics (OAD), statins and aspirin). ACS was defined according to electrocardiographic changes, serial increases in necrosis cardiac biomarkers, or documented coronary artery disease. Patients were diagnosed with ST segment elevation myocardial infarction (STEMI) or unstable angina pectoris/negative ST segment elevation myocardial infarction or (UA/NSTEMI) using standardized criteria. Conventional classification of CVRFs was used [9]. The emergency output mode were exit at home after emergency supervision or transfer to other service (cardiology). Major adverse cardiac events (MACE), were also recorded, as these are the most commonly used composite endpoints in cardiovascular research and were defined in our study as effectiveness outcomes, which included death, major arrhythmia, major conduction disorders and acute pulmonary edema.

### Data collection/measurement

The data register was collected at hospital discharge, in compliance with international guidelines and national laws and regulations. All necessary regulatory submissions were performed in accordance with local regulations, including local data protection regulations. Crude prevalence rates (CPRs) of ACS were calculated based on the Tunisian National Institute of Statistics and expressed in 100,000 inh [10]. The age-standardized prevalence rate (ASR) per 100,000 person-years (PY) was calculated among the world standard population according to the WHO 2013 report.

### Bias

To minimize information bias, we performed training with participating physicians and their staff. Trained physicians and staff in each emergency department verified the data collected and sought to minimize missing data. Authorized secretaries collected and verified data with Statistical Package for Social Science (SPSS) software version 21.0.

### Study size

According to an exploratory study [8], a diagnosis of ACS was confirmed in 38% of chest pain unit patients managed in a Tunisia ED. To have an accuracy of 3%, we had to include at least 1005 patients with nontraumatic acute chest pain. Considering a missing data rate of 10% according to local experience, the target number of subjects to include was 1105.

### Quantitative variables

Age groupings were chosen as young adult (30-40 years), adult (40-60 years) and elderly (> 60 years). Age correlated with sex was defined as men over 55 and women over 65. Medians and inter quartile ranges (IQRs) are used to describe durations.

### Statistical methods

Data were analyzed with SPSS version 21.0. Descriptive, univariate and multivariable statistical analyses were performed.

Categorical variables (age group, sex, cause of chest pain and CVRFs) are presented as counts and percentages. Significant risk factors related to ACS in the univariate analysis with p value of 0.20 were included in the multivariable logistic regression model. The included variables in the first model were age correlated with sex, vascular family history, vascular personal history, coronary personal history, treated hypertension, treated type 2 diabetes, active smoking, treated dyslipidemia and obesity. In the second model, we included the sum of CVRFs as an ordinal variable. The results are expressed as odds ratios (ORs) with 95% confidence intervals (95% CIs). Linear interpolations and Spearman’s rho coefficient were determined to assess the relationship between the number of CVRFS and the risk of ACS.

## Results

### Participants

We included 1173 admissions for ACP; of these, 566 were diagnosed as ACS (49.7%; 95% CI: 46.7– 52.6%). UA/NSTEMI represented 74.2% of all ACS cases.Four patients were excluded from the analysis because they were less than 30 years of age.

### Descriptive data

**Fig. 1:**
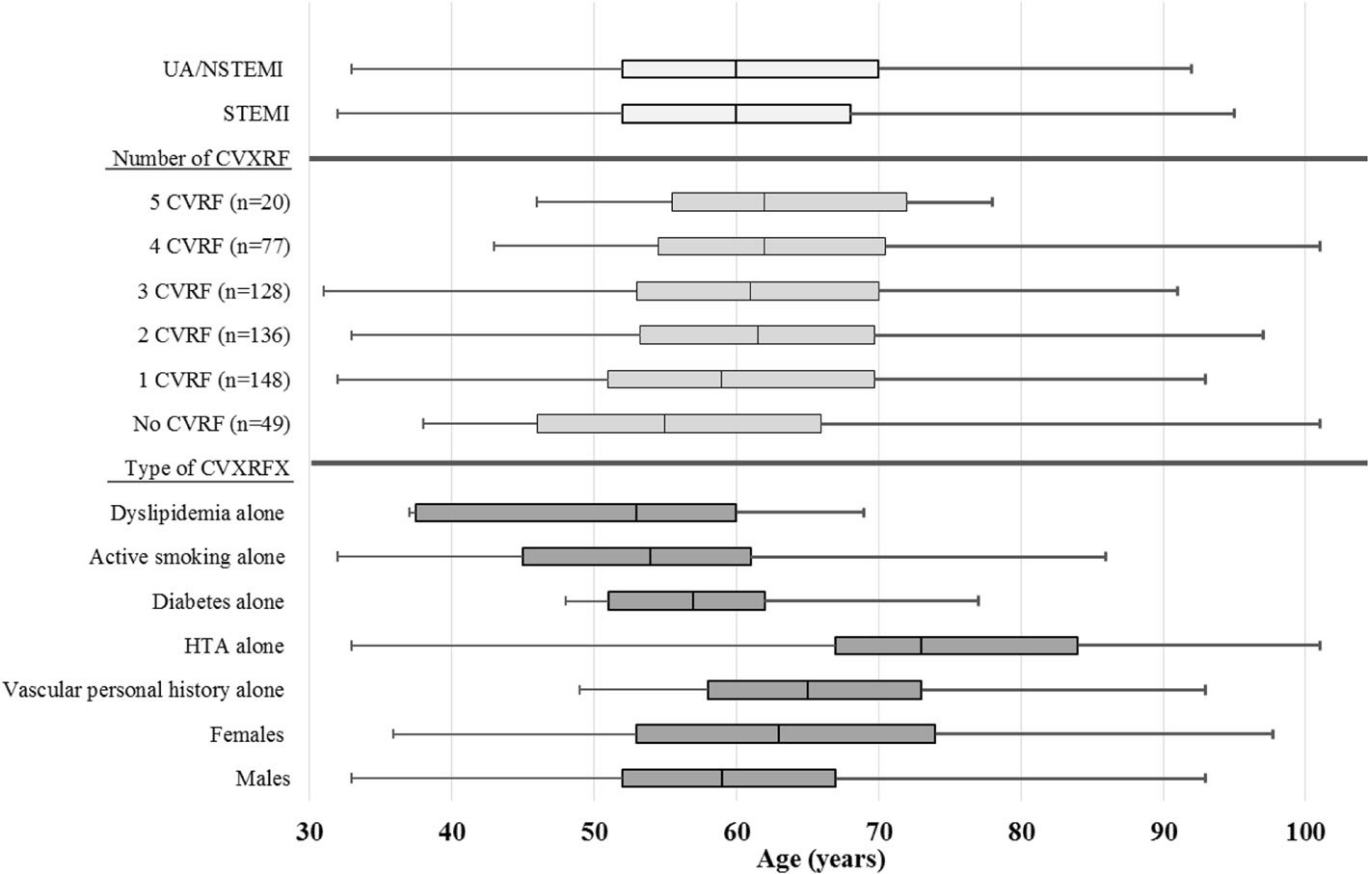
Age distribution by ACS and CardioVascular Risk Factors (CVRFs) subgroups (quartile range (years)). Legend: The median age was equal among patient having STEMI and UA/NSTEMI (60 years (IQR 52-68) and 60 years (IQR 52-70), respectively); it was 55 years (IQR 46-66) among patients with no CVRFs and 62 years in those having five CVRFs

Males represented 67.4% of patients with ACS (*p* < 0.001). The sex ratio (Men/Women) was 3.55 among patients diagnosed with STEMI (Table 1). The median age of ACS patients at admission was 60 years (IQR 52–70); the median age was 63 years for women (IQR 53–74) and 59 years (IQR 52–67) for men (*p*=0.003). According to the CVRFs, the median age at ACS was 54 years (IQR 45-61) among active smokers and 73 years (IQR 67-84) in patients treated for hypertension (Fig 1).

**Table 1:** Chest pain characteristics (*n* = 1173): February– September; 2015

	N	%	Age (years) Median (QR)	Sex-ratio
All chest pain	1173	100	58 (49–68)	1.81
Cause of chest pain				
Cardiac *n* (%)	589	51.71		
Acute coronary syndrome	566	49.69	60 (52–70)	2.07
STEMI	146		60 (52–68)	3.55
UA/NSTEMI	420		60 (52–70)	1.75
Aortic dissection	2	0.18	72	2
Pulmonary embolism	7	0.61	75 (34–80)	0.4
Pericarditis/Tamponade	14	1.23	52 (39–67.5)	2.5
Non-cardiac causes *n* (%)	550	48.29		
Pneumothorax	9	0.79	34 (31–46)	8
Pleurisy	15	1.32	61 (55–78)	1.5
Osteochondritis	35	3.07	62 (58–75)	1.69
Neuralgia of intercostal nerve	149	13.08	53 (45–63.75)	2.36
Digestive pathology	45	3.95	59 (46–73.5)	1.05
Psycho-pathologic disease	106	9.31	51 (39.75–60)	1.08
Irrelevant cause	191	16.77	58 (48–71)	1.58

*QR* quartile range, *UA/NSTEMI* unstable angina/non-ST-segment-elevation myocardial infarction, *STEMI* ST segment elevation myocardial infarction

Legend: The half of patient consulting emergency departments for chest pain were diagnosed as acute coronary syndrome especially UA/NSTEMI

### Outcome data

**Fig. 2:**
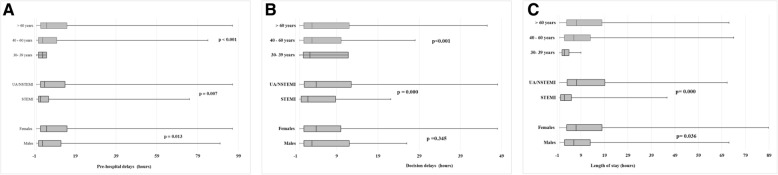
Delays of ACS management in Emergency Departments (EDs) (Tunisia). **a** The median duration between chest pain onset and EDs arrival was 2 h (IQR 2-4 h) for men and 3 h (IQR 2-4 h) for women (*p*= 0.013). This median duration was higher for the elderly [3 h (IQR:2-4 h)] than for younger patients [2 h (IQR:2-4 h)] (*p*=0.007) and among patients with UA/NSTEMI [2 h (IQR 1-4 h)] compared to STEMI [2 h (IQR1-3 h)] (*p* <0.001). **b** The median decision time (duration between ED admission and starting treatment) was 4 h (IQR: 2- 8 h) for all subgroups, for men and women (*p*=0.230). This duration increased with age to 3 h (IQR 2-5h) for 30 - 39-year olds, 4 hours (IQR 2-8 h) for 40 – 60-year olds, and 4 h (IQR 2-10 h) for the elderly (*p*<0.001). The median decision time was 2 h (IQR 0:10-8:30 h) for STEMI and 4 h (IQR 1:00-12:30 h) for UA/NSTEMI patients. **c** The median ED length of stay (LOS) was 1 hour (IQR 0-1) for regional hospitals and 8 hours (h) (IQR 4-18 h) for university EDs (*p*<0.001). The LOS in the ED was higher among woman (7 h; IQR: 3- 18 h) than men (6 h; IQR 2-13 h) (*p*= 0.036). The LOS increased significantly with increasing age; it was 4 hours (IQR: 2-9 h) for patients aged 30-39 years, 6 hours (IQR 2-13 h) for the 40 - 60 years group, and increased to 7 hours (IQR3-18 h) for patients over 60 years (*p*<0.001). Patients with STEMI had a short median LOS (2 h; (0-5 h) compared to those with UA/NSTEMI [8 h (IQR: 4-20 h)] (*p*<0.001)

Emergency medical service transportation was used in 11.9% of cases then 88.1% of patients reached the hospital using their own means of transport. Two hours was the median duration between chest pain onset and ED arrival (IQR: 2-4 h). Pre-hospital delays, starting treatment delays and length of stay were significantly shorter in men, in STEMI cases and among younger patients (Fig 2). Transfer to the Cardiology Intensive Care Unit was performed in 73.9% and 38.3% of STEMI and UA/NSTEMI cases, respectively (Table 2). Forty-three percent of NSTEMI patients were discharged home after surveillance at emergency departments.

**Table 2:** Acute coronary syndrome management according to all ACS, STEMI or UA/NSTEMI

	All ACS (*n*= 566)	STEMI *(n=146)*	UA/NSTEMI *(n=420)*	*p*
			
Duration between chest pain onset and ED arrival : median (IQR) (hour)	2 (1-4)	2 (1-3)	2 (1-4)	0.000
Duration between ED admission and treatment starting: median (IQR) (hour)	3 (1-11)	2 (0.33-8.7)	4 (1.12-12.5)	0.000
ED length of stay: median (IQR)	6 (2.75-16)	2 (0.5-5)	8 (4-20)	0.000
Medications				
β blockers	66 (14.6)	4 (3.6)	62 (18.1)	0.000
Converting enzyme inhibitors	82 (18.3)	11 (9.9)	71 (21.0)	0.009
In hospital management of ACS				
Thrombolysis *n* (%)	41 (7.2)*	37 (25.3)**	4 (0.95)	0.000
Streptokinase	21	19	3	
Alteplase	20	18	1	
Administered treatment: *n* (%)				
Aspirin	499 (91.9)	133 (97.1)	366 (90.1)	0.010
Clopidogrel	392 (74.1)	124 (89.9)	268(68.5)	0.000
LMWH (HNF)	254 (49.9)	84 (64.6)	170 (44.9)	0.000
Outcomes				
MACE	63(11.1)	27 (20.0)	36 (9.1)	0.001
Deaths	7	3	4	0.27
Cardiogenic shock	8	4	4	0.09
Acute Pulmonary Oedema	43	15	28	0.11
Arrhythmia	18	12	6	0.000
Others (conduction disorders)	9	7	2	0.26
Transfer to Cardiology Intensive Care Unit	269	108 (73.9)	161 (38.3)	0.000

*59 missing values; **20: missing values

*ACS* Acute coronary syndrome, *UA/NSTEMI* unstable angina/non-ST-segment-elevation myocardial infarction, *STEMI* ST segment elevation myocardial infarction, *IQR* interquartile range. *LMWH* Low Molecular Weight Heparin. *MACE* Major adverse cardiac events

### Major Adverse Cardiac Events (MACE)

We reported 27 cases of MACE (20.0%) in patients with STEMI and 36 in patients with UA/NSTEMI (9.1%), representing 11.8% in all ACS cases. Seven cases of sudden cardiac arrest were reported (three cases among STEMI patients and four among UA/NSTEMI patients); five resulted in premature death (71.4%) (Table 2).

### Main results

**Fig. 3:**
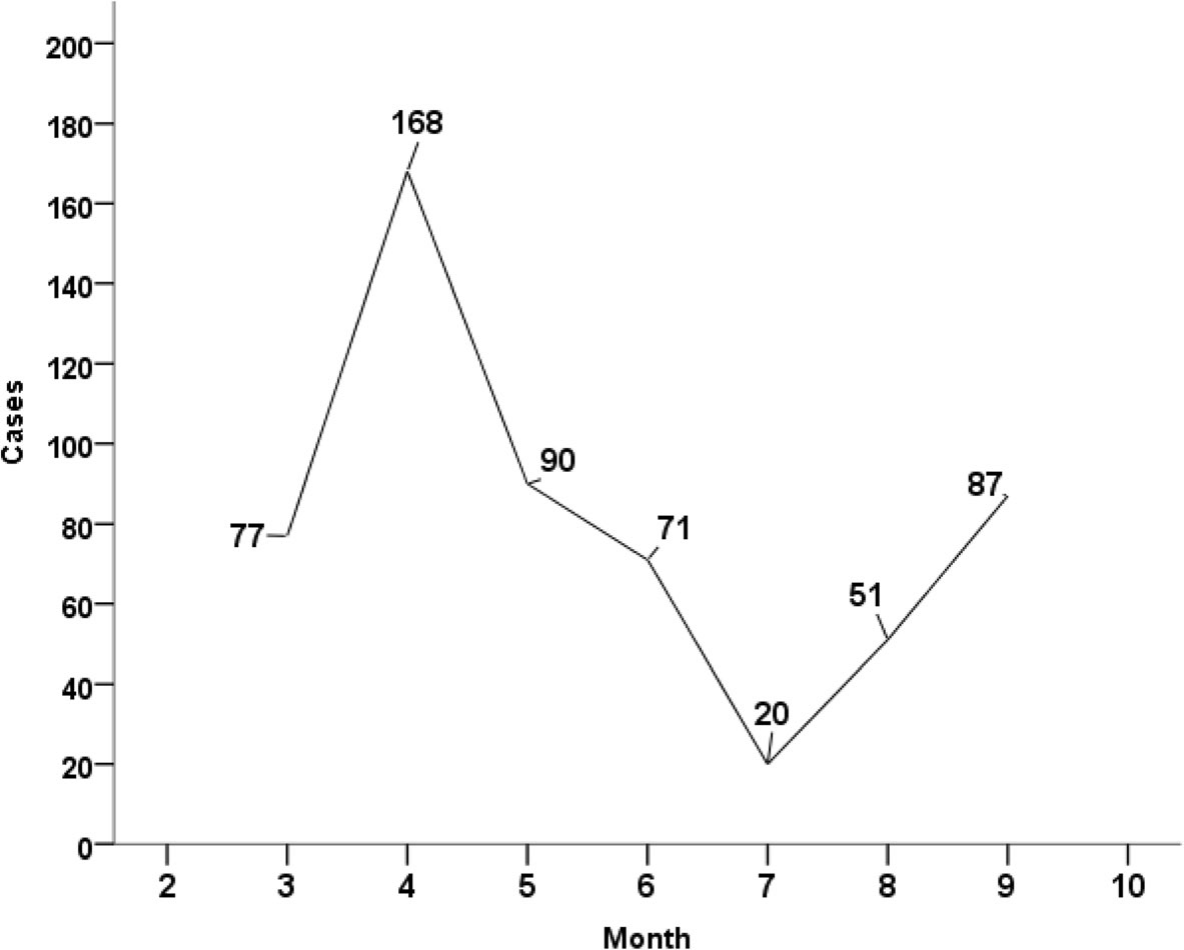
Monthly distribution of acute coronary syndrome (Tunisia, 2015). Legend: Significant variations were observed in the monthly ACS distribution, with the highest in April (*n*=168) and the lowest in July (*n*=20) (*p*=0.001)

Significant variations were observed in the monthly distribution of ACS, with the highest value in April (*n*=168) and the lowest in July (*n*=20) (*p*=0.001) (Fig 3). The CPR was 53.42 per 100 000 inh. The CPR varied from 4.60 to 74.25 and to 175.54 per 100 000 inh among patients aged less than 40 years, 40-60 years old and over 60 years, respectively (*p*< 0.001). The SPR was 69.97/100 000 PY and was higher in men (96.24/100 000 PT) than in women (43.70/100 000 PY) (*p* < 0.001) (Table 3).

**Table 3:** Crude and age-standardized prevalence rates of acute coronary syndrome

	CPR/100000 inh	r	ASR/100,000 PY
All	53.42		69.97
Gender			
Male	86.6		96.24
Female	38.62^*^		43.70
Age groups			
< 40 years	4.60	0.64^*^	
40–60 years	74.25		
> 60 years	175.54		

*CPR* Crude prevalence rate, *r* Standardized coefficient, *ASR* Standardized prevalence rate, *PY* Person year

Missing value for age (*n* = 2). *: *p* < 0.000;

Legend: Prevalence rates were higher in men and population aged more than 60 years

### Distribution of CVRFs and relationship with ACS

**Fig. 4:**
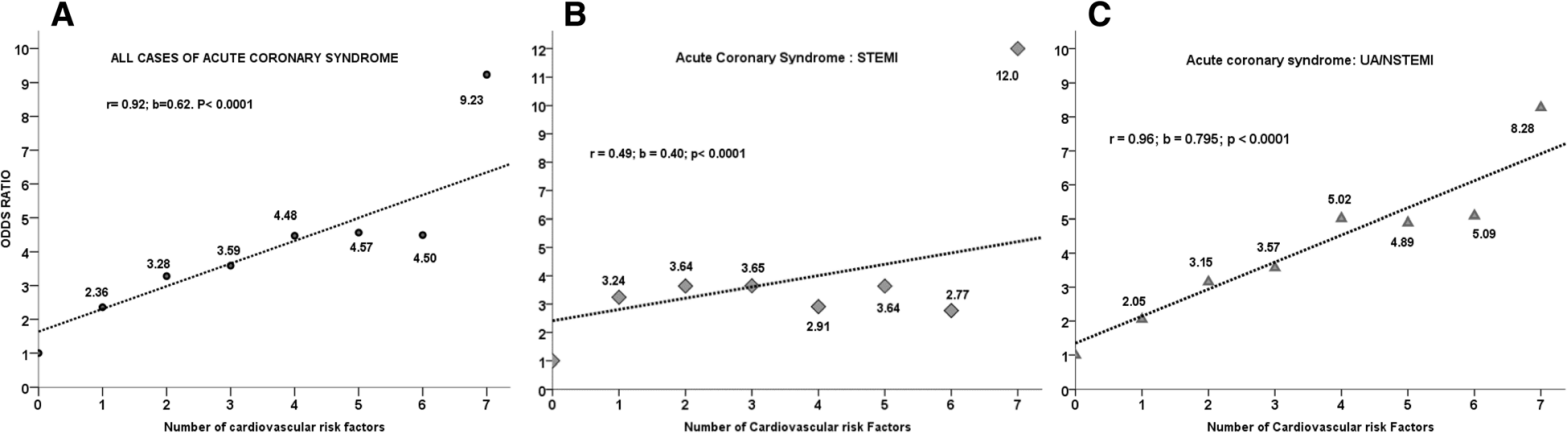
Curve estimation of linear regression for predicting odds ratios through the number of cardiovascular risk factors according to ACS, STEMI or UA/NSTEMI. Legend: **a**: For all cases of acute coronary syndrome: a significant and strong linear relation was established between the number of cardiovascular risk factors and the OR values (r =0.92; b = 0.62; *p* <0.0001), **b**: For UA/NSTEMI linear relation was stronger with (r = 0.96; b =0.795; *p* <0.0001). **c**: For STEMI This linear relation was lower with (r =0.49; b = 0.40; *p* <0.0001).

Hypertension, active smoking and type 2 diabetes were the most reported modifiable CVRFs among patients with ACS (53%). In the multivariable analysis, CVRFs related to STEMI were age correlated to gender (OR:2.55 [95% CI: 1.61-4.06]) and active smoking (OR 2.51 [95% CI:1. 36 - 3.84]). The CVRFs related to UA/NSTEMI were age correlated to gender (OR: 1.47 [95% CI 1.07-2.02]), coronary personal history (OR 2.55 [95% CI 1.79 - 3.64]) and type 2 diabetes (OR 2.34 [95% CI 1.67 -3.28]) (Table 4). A significant and high linear relation was established between the number of CVRFs and the risk of developing ACS (*r* =0.92; b = 0.62; *p* <0.0001), especially with UA/NSTEMI (r = 0.96; b =0.795; *p* <0.0001). The odds ratio of developing UA/NSTEMI was equal to the number of CVRFs +1 (Fig 4).

**Table 4:** Distribution of conventional cardiovascular risk factors according to all ACS, by STEMI or UA/ NSTEMI

	ALL ACS (*n* = 566)		STEMI (*n* = 146)		UA/NSTEMI (*n* = 420)	
Variables	N (%)	OR* [CI95%]	N (%)	OR** [CI95%]	N (%)	OR*** [CI95%]
Cardiovascular risk factors type						
Not modifiable CVRF			146		420	
A/Age correlated with gender	336 (59.4)	1.71 [1.29–2.27]^d^	90 (61.6)	2.55 [1.61–4.06]^d^	243 (57.9)	1.47 [1.07–2.02]^e^
B/Vascular personal history	255 (45.1)		35 (24.0)		210 (50.0)^a^	
Coronary personal history	210 (37.1)	1.87 [1.34–2.59]^d^	24 (16.4)	0.64 [0.36–1.14]	184 (43.8)^a^	2.55 [1.79–3.64]^d^
Peripheral arterial history	38 (6.7)		6 (04.1)		31 (7.4)	
Stroke	50 (8.8)		9 (06.2)		40 (09.5)	
Revascularization	145 (25.6)		13 (08.9)		131 (31.2)^a^	
Modifiable and direct CVRF						
C/Treated HTA	300 (53.0)	1.23 [0.91–1.68]	65 (44.5)	1.08 [0.66–1.75]	232 (41.8)	1.26 [0.90–1.75]
D/Treated Diabetes type 2	248 (43.8)	2.04 [1.49–2.80]^d^	51 (34.9)	1.30 [0.76–2.21]	195 (52.4)^b^	2.34 [1.67–3.28]^d^
E/Active smoking	262 (46.3)	1.50 [1.14–2.00]^e^	81 (55.5)	2.51 [1.63–3.84]^d^	178 (36.6)^b^	1.18 [0.86–1.60]
F/Treated Dyslipidemia	162 (28.6)	0.81 [0.57–1.15]	29 (19.9)	0.77 [0.43–1.37]	132 (44.1)^b^	0.78 [0.53–1.14]
Modifiable and indirect CVRF						
G/ Obesity	147 (26.0)	1.18 [0.84–1.65]	34 (23.3)	1.02 [0.58–1.73]	111 (39.6)	1.21 [0.84–1.74]
Patients treated by CEI	192 (33.9)	0.72 [0.53–0.97]^d^	35 (24.0)	0.56 [0.33–0.93]^d^	157 (37.4)^a^	0.77 [0.52–1.14]
Sum of CVRF						
0	39 (6.9)	1,00	10 (6.8)	1,00	29 (06.9)	1,00
1	105 (18.6)	2,36 [1.52–3.67]^d^	34 (23.3)	3,24 [1.55–6.79]^d^	68 (16.2)	2,05 [1.25–3.38]^d^
2	130 (23.0)	3,28 [2.12–5.08]^d^	37 (25.3)	3,64 [1.73–7.65]^d^	93 (22.1)	3,15 [1.94–5.13]^d^
3	119 (21.0)	3,59 [2.29–5.62]^d^	31 (21.2)	3,65 [1.71–7.80]^d^	88 (21.0)	3,57 [2.17–5.86]^d^
4	96 (17.0)	4,48 [2.77–7.22]^d^	16 (11.0)	2,91 [1.25–6.77]^f^	80 (19.0)	5,02 [2.98–8.44]^d^
5	49 (08.7)	4,57 [2.58–8.08]^d^	10 (06.8)	3,64 [1.39–9.47]^e^	39 (09.3)	4,89 [2.64–9.05]^d^
6	19 (03.4)	4,50 [2.04–9.93]^d^	3 (02.1)	2,77 [0.67–11.36]	16 (03.8)	5,09 [2.21–11.76]^d^
7	6 (01.1)	9,23 [1.79–47.61]^e^	2 (01.4)		4 (01.0)^a^	8,28 [1.45–47.39]^f^

Chi 2 pearson test (STEMI vs UA/NSTEMI): ^a^*p* value < 10^−3^; ^b^:*p* value <0.005

Binary logistic analysis*: All SCA vs All chest pain; **All STEMI vs All chest pain: ***: All UA/NSTEMI. vs All chest pain; ^d^: *p* value < 0.001; ^e^:*p* value < 0.005; ^f^:*p* value < 0.05

*ACS* Acute coronary syndrome, *UA/NSTEMI* unstable angina/non-ST-segment-elevation myocardial infarction, *CVRF* cardiovascular risk factors, *STEMI* ST segment elevation myocardial infarction, *OR* Odds Ratio, *HTA* Hypertension, *CEI* converting enzyme inhibitors

## Discussion

### Key results

We reported that ACS occurred among half of the patients presenting for chest pain in emergency departments in Tunisia. The management of ACS was weak, and the duration between chest pain onset and ED arrival was longer than in developed countries. According to age-standardized prevalence rates, the ACS prevalence was significantly lower than in developed countries. Male predominance is universal. By logistic binary regression, cardiovascular risk factors related to ACS, adjusted for age and gender, were smoking for ACS with STEMI and diabetes and coronary personal history for ACS with UA/NSTEMI. By linear regression, we established a significant and strong relationship between the number of associated cardiovascular risk factors and estimated odds ratios, especially for ASC with UA/NSTEMI. MACE occurred more frequently among STEMI patients.

### Limitations

Several limitations should be considered. Due to the short study duration, the results must be interpreted with caution. It is worth further evaluation of outcomes with a longer follow-up. The private sector dealing with ACS patients was not included, and some patients with other form of ACS (silent MI) were not diagnosed. Therefore, these findings underestimated ACS in the whole Tunisian population.

### Interpretation

Prospective national studies on the epidemiology of ACS are not as frequently reported in developing countries as in Tunisia. In this multicenter study that included fourteen regional and university hospitals, we enrolled 1173 patients consulting the ED for ACP. ACS prevalence, management and relation with CVRFs have rarely been described in our country [11–13]; it was considered necessary to conduct this study to provide the basis for adapted public health strategies and decisions.

The prevalence of ACS was similar to that described by Baccouche et al. [9] in a monocentric study performed in the Monastir ED three years ago. This relatively low Tunisian ACS prevalence can be explained by lifestyle differences and local habits [14]. The UA/NSTEMI predominance in our study was similar to that described by Dilip et al. [15] and El-Menyar et al. [16] and different than the results of Bacci [17]. Gender and age distribution correlated with ACS are widely described in the literature [18, 19]. We have observed that the median age of ACS was 54 years among smokers and 73 years among patients treated for hypertension. Our results were in agreement with those of Dilip et al. [15]. Due to the increased rate of cardiac events in smokers, further efforts for smoking cessation must be performed. The duration between chest pain onset and ED arrival and starting treatment were higher than those described by Mark et al. [20]. These times varied according to gender, age groups and ACS type. Appropriate identification of patients with symptoms of chest pain and early discharge as recommendations should be improved [21–24]. We explained the increasing duration between chest pain onset and emergency arrival with increasing age by the emergence of social loneliness of the elderly in Tunisia [11, 14]. The increasing length of stay in ED with increasing age, was explained by Jin H et al. as related to low education level, co-morbidities, and the total number of discharge medications for elderly patients [25]. We observed a significantly longer duration between ED admission and treatment among patients diagnosed as UA/NSTEMI versus STEMI. Mark et al. established that these delay parameters were not influenced by socio-demographic characteristics, vascular family history or the ACS subgroup (STEMI or UA/NSTEMI); they considered that this duration is influenced by the knowledge of general practitioners and a better ambulance service [20]. Adherence to international guidelines, in our cohort, was largely less than that described by Gregory J. Dehmer et al. [26] in the United States and by Prashanth Panduranga in Oman [27]. The SPR was 69.97 /100 000 inh in our study, which is lower than the rates reported in developed and rich countries [28–31] and in Arabian Gulf countries, in which Al-Lawatiet et al. described a rate of 779 and 674 per 100,000 PY for men and women, respectively, in 2007 [29]. The variability between countries might be explained by several factors such as socio-demographic conditions, population education levels, health politics implications and, especially, nutritional habits. We have gathered literature evidence of a predominance of men [18]. Women presenting with ACS are older than men regardless of the increasing risk with increasing age in the two genders [32]. We have determined that the risk of ACS is highly correlated to the number of conventional risk factors. Eighteen percent of our patients with ACS had at least one conventional risk factor. In our study, active smoking was the most modifiable CVRF observed (55.5%) among STEMI cases and is directly associated to ACS, especially to STEMI; similar results were observed by El-Menyar et al. [16]. Smoking was also found to be a high risk factor by Kastorini et al. and Notara et al. [33, 34]. Diabetes mellitus was the most modifiable CVRF present (52.4%) among UA/NSTEMI cases. Diabetes was associated with all ACS and to UA/NSTEMI. Our results are consistent with literature data [15, 35, 36]. Ahmadi et al. concluded that patients with metabolic syndrome have a significantly greater prevalence, severity, and prognosis of coronary artery disease [37]. Chalghoum et al. observed a metabolic relationship between endothelin-1 and ACE inhibitors among Tunisians with a coronary personal history [38]. Singh et al. reported a significant association between ACE inhibitors and decreased in-hospital remyocardial infarction risk.

### Generalizability

The implementation of an ACS register will give us a method for monitoring prevalence trends and management, providing important information for health policy decisions. Moreover, there is a need to increase adherence to international guidelines to improve patient outcomes. These findings should be confirmed by other national registries.

## Conclusions

Our results showed a high prevalence of ACS in a Tunisian population presenting to the emergency department for acute chest pain, with a close relation with CVRFs. Efforts for a public awareness campaign and training program for ED physicians that have improved ACS management in Tunisian EDs should be maintained and expanded.

## Abbreviations

ACE inhibitors: converting enzyme inhibitors
ACS: Acute coronary syndrome
ASR: Age-standardized prevalence rate
CPRs: Crude prevalence rates
CVRFs: Cardiovascular risk factors
EDs: Emergency departments
Inh: Inhabitants
MACE: Major adverse cardiac events
OAD: Oral antidiabetics
STEMI: ST segment elevation myocardial infarction
UA/NSTEMI: Unstable angina/non-ST-segment- elevation myocardial infarction
